# “The peripheral perfusion index discriminates haemodynamic responses to induction of general anaesthesia”

**DOI:** 10.1007/s10877-023-01035-z

**Published:** 2023-06-08

**Authors:** Jakob Højlund, David René Petersen, Marianne Agerskov, Nicolai Bang Foss

**Affiliations:** 1 Department of Anaesthesiology, Hvidovre University Hospital, Capital Region, Denmark; 2 Currently Department of Anaesthesiology, CKO, Rigshospitalet, Capital Region, Denmark

**Keywords:** Induction period, General Anaesthesia, Circulatory monitoring, Peripheral perfusion index, Non-invasive monitoring

## Abstract

Induction of general anaesthesia is often accompanied by hypotension. Standard haemodynamic monitoring during anaesthesia relies on intermittent blood pressure and heart rate. Continuous monitoring systemic blood pressure requires invasive or advanced modalities creating a barrier for obtaining important information of the circulation. The Peripheral Perfusion Index (PPI) is obtained non-invasively and continuously by standard photoplethysmography. We hypothesized that different patterns of changes in systemic haemodynamics during induction of general anaesthesia would be reflected in the PPI. Continuous values of PPI, stroke volume (SV), cardiac output (CO), and mean arterial pressure (MAP) were evaluated in 107 patients by either minimally invasive or non-invasive means in a mixed population of surgical patients. 2 min after induction of general anaesthesia relative changes of SV, CO, and MAP was compared to the relative changes of PPI. After induction total cohort mean(± st.dev.) MAP, SV, and CO decreased to 65(± 16)%, 74(± 18)%, and 63(± 16)% of baseline values. In the 38 patients where PPI decreased MAP was 57(± 14)%, SV was 63(± 18)%, and CO was 55(± 18)% of baseline values 2 min after induction. In the 69 patients where PPI increased the corresponding values were MAP 70(± 15)%, SV 80(± 16)%, and CO 68(± 17)% (all differences: p < 0,001). During induction of general anaesthesia changes in PPI discriminated between the degrees of reduction in blood pressure and algorithm derived cardiac stroke volume and -output. As such, the PPI has potential to be a simple and non-invasive indicator of the degree of post-induction haemodynamic changes.

## Purpose

Haemodynamic monitoring during general anaesthesia (GA) traditionally relies on simple and readily available parameters such as blood pressure and heart rate. Most patients will only have intermittent non-invasive monitoring of blood pressure. During the induction phase an abrupt reduction in blood pressure is often seen, with possible deleterious consequences for organ perfusion [1, 2]. The availability of continuous monitoring of systemic haemodynamics is limited by cost and scarcity of equipment and personnel.

The peripheral perfusion index (PPI) is obtained non-invasively by photoplethysmography – ubiquitously present in the perioperative setting. The PPI is a simple ratio describing the proportion of pulsatile to non-pulsatile signal attenuation. No specialized equipment is needed as the PPI is inherent to photoplethysmography, although not all pulse-oximeters are by default set up to display PPI. The PPI in awake patients is right skewed and dominated by sympathetic tone [3, 4]. Conversely, during GA (with induced sympatholysis) changes in cardiac stroke volume (SV) becomes the major determinant of changes in PPI [4, 5]. Very little evidence exists regarding the feasibility of PPI as a monitor during the induction of GA. Presumably, the net effect will be a composite of two opposing mechanisms: Sympatholysis increasing PPI, with decreased SV/CO causing the opposite effect.

The present study was designed to explore the clinical utility of the non-invasively obtained peripheral perfusion index to detect cardiovascular compromise during induction of general anaesthesia. We hypothesized that during induction of GA changes in systemic haemodynamics in the form of mean arterial pressure (MAP), cardiac stroke volume (SV) and -output (CO) would be reflected in the PPI, albeit in a complex manner. Thus, a predominant vasodilatory response might be differentiated from a response dominated by decreased CO.

To examine the ability to of the PPI to describe different haemodynamic responses during induction of GA, we investigated, in a prospective mixed cohort of patients undergoing surgery under GA, associations between acute changes in PPI, MAP, SV, and CO during the first minutes of induction.

Furthermore, we wanted to investigate whether baseline PPI could predict haemodynamic instability during induction, as well as offer baseline data of PPI in a mixed cohort of surgical patients.

## Methods

### Study population

For this prospective observational study we included 107 patients scheduled for acute or elective surgery at our institution. Patients were included when one of the investigators were available and logistics permitted. If no vasopressor or inotrope is administered a mean decrease in MAP 25% after induction is likely [6, 7]. However, no data was available to infer any knowledge of PPI during induction. Hence, we set out to include at least 100 to have a statistically robust data set. All patients gave oral and written consent to participate.

### Study setting

All patients were monitored with continuous blood pressure. Either by arterial catheter (n = 49) dependent on local protocol or by continuous non-invasive blood pressure monitoring by a CNAP module (CNSystems, Graz, Austria). Calculations of SV and CO from raw BP data were performed by a LiDCO Unity monitor (LiDCO, London, UK). A Masimo Radical 7 pulse oxymeter probe (Masimo, Irvine, CA, USA) was placed on the third finger of the right hand and shielded from ambient light, providing continuous recordings of PPI.

After 5 min of baseline recording induction of anaesthesia was performed in the supine position using propofol and remifentanil. Elective or rapid sequence induction, airway management, and drug dosage was at the discretion of the attending anaesthesiologist. All measurements were recorded at 2 min post-induction, after which the study was considered terminated. No vasopressor or inotropic agent was administered routinely during induction but could be used if deemed necessary for patient safety before the 2 min post induction measurements, which was the case in three patients. This setup was chosen to mimic the clinical reality of intermittent non-invasive blood pressure measurements by brachial cuff for the vast majority of patients.

### Primary outcomes

Changes in MAP, SV, and CO stratified by changes in PPI, both by quartiles and dichotomized by PPI decreasing vs. increasing.

### Secondary outcomes

Baseline correlations between the investigated variables. Predictive power of baseline PPI for post induction haemodynamic instability.

### Data analysis

Data from the LiDCO monitor (MAP, SV, CO, HR) was exported and analyzed using LiDCOviewPRO V1 (LiDCO, London, UK). Data from the MASIMO monitor was exported to excel spreadsheet. The sampling periods were 30 s one minute before induction and 30 s 2 min after induction. The mean values of the variables during the sampling periods were calculated. Relative changes for SV, CO, MAP, and PPI were calculated.

### Statistics

Distribution of outcome data were tested with the Kolmogorow-Smirnov test for normality and inspected graphically with qq-plots. Normal distributed data (MAP, SV, HR, and CO) are reported as mean(± st.dev.) and data with non-normal distribution (PPI) as median[IQR].

Correlation between variables was evaluated by the Pearson correlation test or the Spearman Rank-order correlation where appropriate. Differences of means between groups were evaluated by T-test.

Analyses were performed using SPSS version 25 statistical software (IBM, Armonk, NY, USA) and a 2-sided p-value < 0,05 was considered statistically significant.

## Results

Basic characteristics of the cohort are presented in Table 1.

**Table:** Table 1

Patient characteristics	
Patients, n	107
Female, n (%)	58 (54)
Age, years ± SD	60 ± 18
Height, cm ± SD	171 ± 10
Weight, kg ± SD	82 ± 19
BMI, kg/m^2^ ± SD	28 ± 6
ASA physical status, n (%)	
1	15 (14)
2	60 (56)
3	32 (30)
4	0 (0)
Emergency Surgery, n (%)	30 (28)
Co-morbidities, n (%)	
Hypertension	49 (46)
Ischemic heart disease	10 (9)
Congestive heart failure	4 (4)
COPD	14 (13)
Hypothyreosis	5 (5)
Atrial Fibrillation	8 (7)
Diabetes mellitus	8 (7)
Cardiovascular Medication, n (%)	
Hydrochlorthiazide	17 (16)
ACE-I / ARB	30 (28)
Calcium channel blockers	13 (12)
B-receptor antagonists	8 (7)

### Baseline hemodynamics

At baseline PPI was 1,7[1,0–3,8]%, MAP was 99(± 19)mmHg, SV was 99(± 34)mL, and CO was 7,3(± 2,8)L/min.

### Baseline correlations

Pre-induction PPI correlated weakly and negatively with MAP (r = -0,22), p < 0,05. No correlation with CO or SV was found. No correlation with age was found. PPI did not differ between patients with known hypertension and those without. No significant effect of treatment with ACE-inhibitors or ACE receptor blockers was found.

### Predictive value of PPI

No correlation between baseline PPI and hemodynamic response to induction was found.

### Effects of induction, total cohort

2 min after induction PPI was 2,9[1,5 − 4,8]%, MAP was 65(± 17)mmHg, SV was 74(± 36)mL, and CO was 4,6(± 2,6)L/min. These values correspond to relative changes of + 36[-21–105]%, -35(± 16)%, -26(± 18)%, and − 37(± 19)%.

### Stratifying hemodynamic response to induction into quartiles based on PPI response to induction (Table 2)

**Fig. 1:**
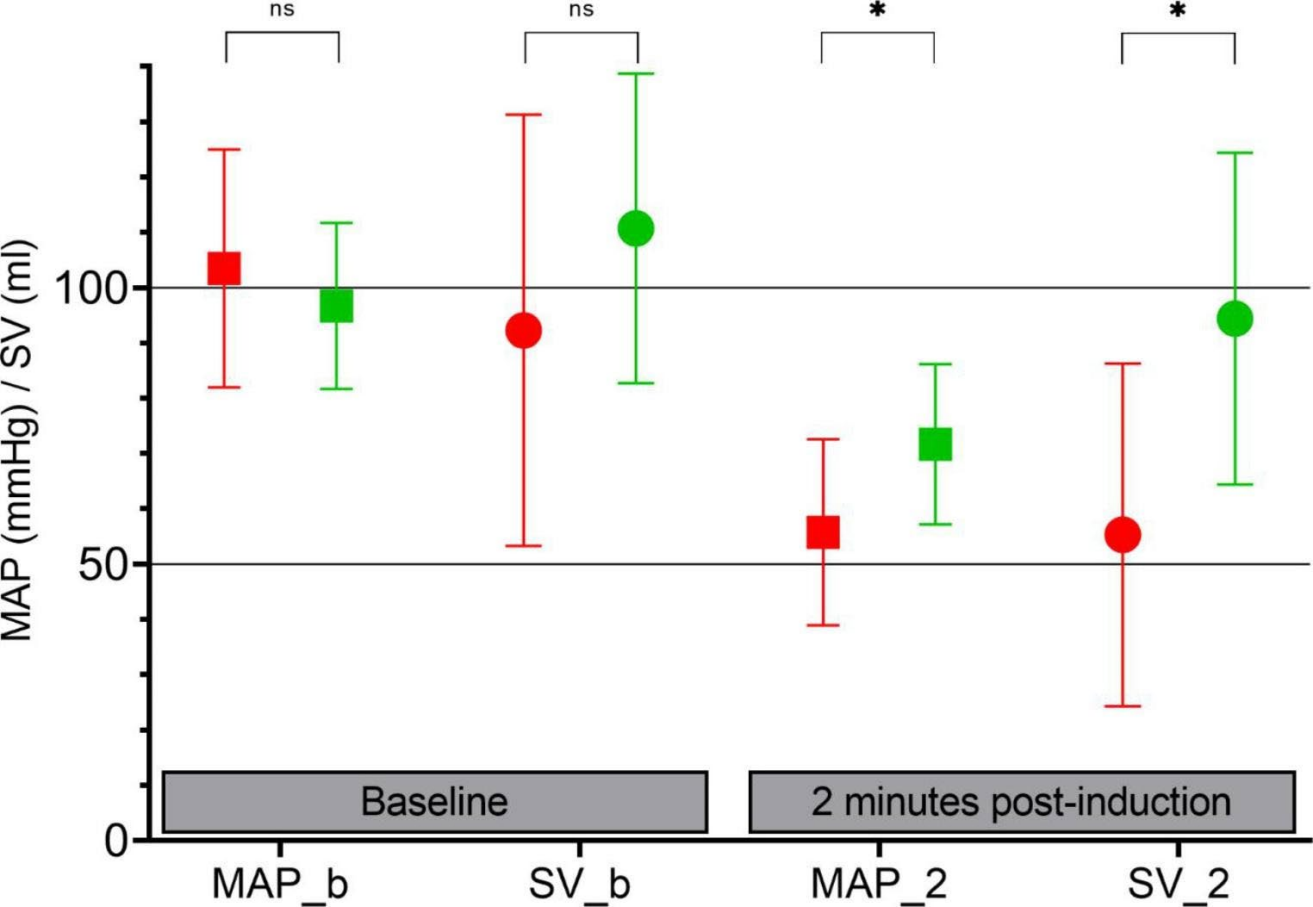
Effect of Induction by 1st and 4th ΔPPI Quartiles Effect of induction on MAP and SV discriminated by lowest ΔPPI Quartile (red) or highest ΔPPI Quartile (green). Absolute values at baseline and 2 min post-induction Squares: Mean arterial pressure at baseline (MAP_b) and at 2 min post-induction (MAP_2) Circles: Cardiac stroke volume at baseline (SV_b) and at 2 min post-induction (SV_2) ns = p > 0.05 ✱ = p < 0.001 (T-test)

To better describe the breadth of circulatory changes within the cohort we stratified the patients based on PPI response to induction, where the first quartile was the largest decrease in PPI at 2 min, whereas the fourth was the largest increase in PPI at 2 min. Doing this we found a gradual increase in cardiovascular compromise, the largest in the 1st quartile (PPI range 0–78% of baseline) with MAP 54(± 13)%, SV 59(± 16)%, and CO 49(± 16)% of baseline values, and the least in the 4th quartile (PPI range 206–720% of baseline) with MAP 75(± 16)%, SV 86(± 16)%, and CO 71(± 18)% of baseline values (Fig. 1). Pairwise comparison found MAP, SV, and CO significantly different between first and second quartiles, and SV and CO significantly different between third and fourth quartiles, p < 0,05.

### Stratifying hemodynamic response to induction by qualitative PPI response to induction (Table 2; Fig. 2)

In the PPI decreasing group (n = 38) MAP was 57(± 14)%, SV 63(± 18)%, and CO 55(± 18)% of baseline values. In the group with PPI stable or increasing (n = 69) MAP was 70(± 15)%, SV 80(± 16)%, and CO 68(± 17)% of baseline values. All responses differed significantly between the 2 groups, p < 0,001.

**Table 2:** , Effect of induction on PPI and cardiovascular variables, percentage of pre-induction values

2 min post-induction values relative to baseline, stratified by PPI quartiles						
	n	PPI, %	MAP, %	SV, %	CO, %	HR, %
Q1	27	58[31–65]	54(± 13)	59(± 16)	49(± 16)	82(± 10)
Q2	26	104[89–120]	66(± 14)	76(± 14)	65(± 16)	85(± 15)
Q3	27	167[149–180]	66(± 14)	75(± 17)	67(± 19)	89(± 13)
Q4	27	276[225–333]	75(± 16)	86(± 16)	71(± 18)	83(± 13)
2 min post-induction values relative to baseline, stratified by dichotomized PPI response						
	n	PPI, %	MAP, %	SV, %	CO, %	HR, %
PPI decreasing	38	65[40–81]	57(± 14)	63(± 18)	55(± 18)	84(± 13)
PPI Increasing	69	175[138–233]	70(± 15)	80(± 16)	68(± 17)	85(± 13)

PPI given as median[IQR], all other values given as mean(± st.dev.)

**Fig. 2:**
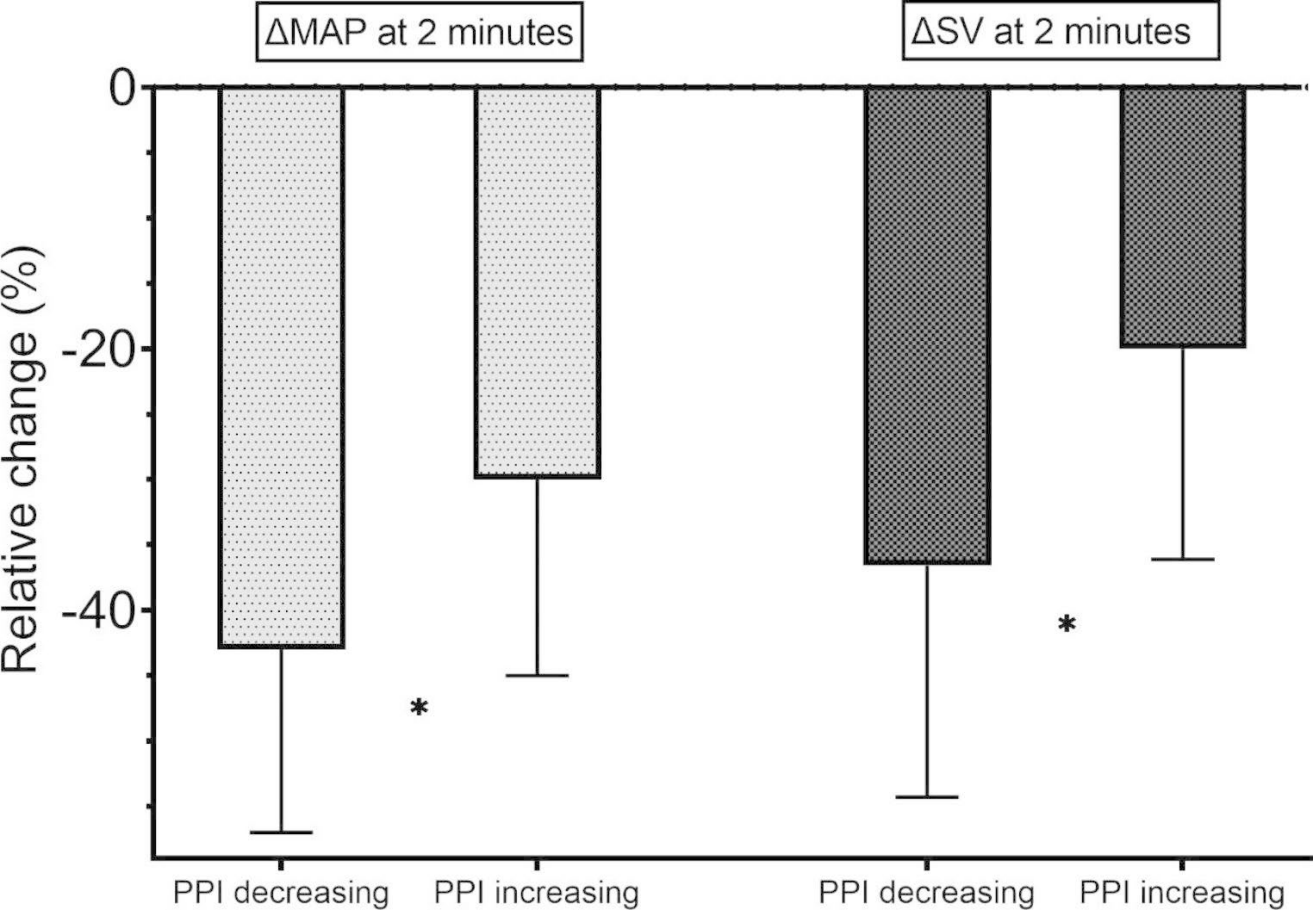
Effect of induction by PPI response (decreasing vs. increasing) Effect of induction on ΔMAP at 2 min and ΔSV at 2 min discriminated by ΔPPI response at 2 min. With PPI increasing the change at 2 min is significantly less. ✱ = p < 0.001 (T-test) Light grey bars: Mean arterial pressure at 2 min post-induction relative to pre-induction Dark grey bars: Cardiac stroke volume at 2 min post-induction relative to pre-induction

## Discussion

During induction of general anaesthesia a range of changes to the circulation happens in a very short span of time, with possible deleterious consequences for organ perfusion [8]: Loss of sympathetic tone leads to decreased afterload and preload, as well as negative ino- and chronotropy, the latter cardiac effects may also reflect a direct effect of the induction agents. The cumulative systemic effects are variable, but decreasing MAP and SV is frequent with modern anaesthetics, albeit with SV maintained to a higher degree than blood pressure [9, 10]. Even so, most patients will only have intermittent non-invasive monitoring of blood pressure, along with heart rate and pulse plethysmography for arterial oxygen saturation. Advanced and continuous monitoring focusing on optimizing systemic blood flow is gaining popularity [11]. However, continuous monitoring of systemic and central haemodynamics is more invasive and/or requires specialized equipment and continuous training [12]. Thus, these modalities will only be offered to a small subset of patients. A potentially large group of patients is subject to “less-than ideal-monitoring” during induction of general anaesthesia – due to lack of resources, unrecognized cardiovascular risk, and/or unanticipated susceptibility to cardiovascular compromise.

The peripheral perfusion index (PPI) is obtained non-invasively by photoplethysmography, ubiquitously present during GA. The PPI in awake patients is predictive of the complication rate in major surgery [13], is a predictor of mortality in severe acute illness [14–16], predicts hypotension during fluid withdrawal in uremic patients [17], and hypotension induced by spinal anesthesia for sectio caesarea [18, 19], as well as being an early predictor of central hypovolemia and compensatory sympathetic activation in healthy volunteers [20, 21]. In high-risk surgical patients the intra-operative PPI is a strong predictor of serious post-operative complications or death [22]. During GA the PPI tracks changes in MAP and SV induced by manipulation of preload [5]. To our knowledge only one previous study have assessed the PPI during induction of anaesthesia [6].

The plethysmographic signal consists of a pulsatile “AC” and non-pulsatile component “DC” with PPI = AC/DC. Sympathetic tone is the major determinant of PPI in the awake person. Indeed, sympathetic block demonstrated by increasing PPI has been described as determinant of successful neuraxial [23] or peripheral [24, 25] block. During general anaesthesia sympathetic outflow is reduced to a very low baseline [26–31], abolishing its dominance over the PPI. Thus, the main determinant of PI becomes SV [4, 5, 32, 33].

Clearly the PPI during the transitional period of induction of anaesthesia is subject to multiple changes from both sympatholysis and alterations of global circulatory parameters. Still, we hypothesized the possibility of.

describing two different patterns of sympatholysis: One dominated by vasodilatation with preserved stroke volume and flow (≈ PPI increasing) and one with decreased cardiac stroke volume dominating (≈ PPI decreasing).

The LiDCO Unity monitor is an uncalibrated device calculating SV from the arterial pulse waveform. The algorithm tracks changes in SV/CO with good reliability [11, 34, 35]. For patients without an arterial cannula, we used the CNAP module to provide a high-resolution continuous trace of the arterial pressure curve to the LiDCO monitor. This technology tracks the intra-arterial pressure curve with good reliability, although tracking may be less precise during induction [36–38].

In this cohort of mixed surgical patients, we found a baseline PPI of 1,7[1,0–3,8]% (median and quartiles), not unlike 1,4% previously reported in healthy volunteers [3]. Mean(± SD) baseline PPI was 2,6(± 2,3) (due to the right-skewed distribution) consistent with a large recent cohort of high risk surgical patient reporting a mean PPI of 2,6(± 2,6) [22].

We did not find baseline PPI to be predictive of hemodynamic instability during induction. It would not be unreasonable to expect a very low PPI (e.g. <0,5%) to be predictive of post-induction instability as a low PPI might suggest a circulation dependent on high sympathetic tone. Most likely our cohort consisted of “too healthy” patients to show this effect, indeed only 4 patients had a very low baseline PPI < 0,5%. This may be due to patients being included only when the investigators were present with sufficient time to setup the equipment, but also due to the fact that acutely ill patients may not be able to give informed consent for a number of reasons. Furthermore, anxiety or pain, may also lead to a low PPI, but not a higher likelihood of post-induction instability. Thus, the negative correlation found between baseline MAP and PPI reflects the effect of sympathetic tone on PPI in the awake patient [4].

On average for the total cohort 2 min after induction PPI increased by 36%, whereas MAP, SV, and CO decreased by 35%, 26%, and 37%, respectively. This is consistent with a recently published study describing an inverse correlation between ∆MAP and ∆PPI during induction [6]. However, our study having a larger and more diverse population, as well as monitoring of cardiac stroke volume and - output, allows for a deeper insight into the mechanisms influencing the PPI. Stratifying the changes in PPI upon induction makes for a more comprehensive image of the relationship between PPI and systemic hemodynamics during induction. Even though PPI on average increases and indices of systemic circulation (MAP, SV, CO) on average decreases, a more compelling narrative appears: Stratifying PPI response in quartiles shows that with larger post-induction increase in PPI an attenuated cardiovascular compromise is seen. In the quartile of highest PPI increase especially SV is preserved, only decreasing by 14% of pre-induction values. Conversely, in the quartile of in the lowest post-induction PPI values marked cardiovascular compromise is seen, with MAP reduced to 54% and SV to 59% of baseline values (Table 2; Fig. 1).

These changes likely reflect different degrees of reduction in cardiac function due to induction of anaesthesia. The highest increase PPI quartile thus have decreased arterial vasomotor tone but largely intact stroke volume contrasting the low PPI quartile with compromised cardiac function – due to sympathetic dependent cardiac function, reduced cardiac filling by venous dilatation, or both. As noted, the major determinant of PPI in the awake patient (i.e. pre-induction/baseline) is sympathetic tone. Upon induction, all things being equal, the PPI will increase as vascular tone decreases. However, as sympathetic tone is decreased changes in SV becomes the major determinant of PPI; with a large post-induction decrease in cardiac function leading to net decrease in PPI, whereas a cardiac function only mildly affected by induction of anaesthesia leads to a net increase in PPI [4].

A recent study using non-invasive monitoring during induction found a stable SV and decreased MAP after induction [9]. The patients differ from those in our study by being much younger (mean 36 vs. 60 years) and healthier (ASA 1–2: 91% vs. 70%). The pattern of preserved SV during induction thus resembles the most stable patients of out cohort; the quartile with the highest increase in PPI during induction.

To increase the clinical usefulness of our results, we dichotomized our data into PPI decreasing vs. PPI stable or increasing (Fig. 2). With PPI decreasing MAP was reduced to 57% and SV to 63% of baseline; with PPI increasing MAP was 70% and SV 80% of baseline. Again, a clear pattern emerges leading to a simple recommendation to the clinician: Observe the changes in PPI during induction of anaesthesia: Should the PPI not increase during induction our data suggests increased risk of compromised cardiovascular function. Thus, when observing a decreasing trend of PPI upon induction of anaesthesia, the provider should be prepared for a higher risk of hemodynamic compromise.

We must point out that algorithm-based SV measurements may be inaccurate during the induction period; this may hold even more true when the pulse waveform is derived from CNAP instead of arterial cannula. However, due to the high temporal resolution of this methodology it enables to obtain some insight, where more accurate but slower methods cannot be used. Thus, these results point a direction for further research into this evolving field of interest.

In summary, in a mixed cohort of surgical patients undergoing general anaesthesia using minimally- or non-invasive monitoring of MAP, SV, and CO we demonstrated the expected effects of induction of anaesthesia with reduction of haemodynamic parameters. When stratifying the patients according to the effect of induction upon PPI it was possible to elicit a plausible physiological background to the observations and to discriminate between trivial and more severe degrees of cardiovascular compromise.
